# The Limits of the Primacy of Morality Hypothesis: Are Global Impressions of Experts Based Mainly on their Competence or Morality?

**DOI:** 10.5334/irsp.643

**Published:** 2023-04-21

**Authors:** Katarzyna Stasiuk, Mariola Paruzel-Czachura, Romuald Polczyk, Józef Maciuszek

**Affiliations:** 1 Institute of Applied Psychology, Jagiellonian University in Krakow, Poland; 2 Institute of Psychology, University of Silesia in Katowice, Poland; 3 Penn Center for Neuroaesthetics, University of Pennsylvania, United States; 4 Institute of Psychology, Jagiellonian University, Poland

**Keywords:** competence, morality, experts, the primacy of morality hypothesis, social perception

## Abstract

According to the primacy of morality hypothesis, moral traits are the most substantial contributor to – and when positive, always contribute positively to – global impressions of others. In three experiments (*N* = 413), we asked participants to form global impressions of the financial advisor (Study 1a), car mechanic (Study 1b), and physician (Study 1c). Contrary to the primacy of morality hypothesis, we showed that when people evaluate experts, they are guided primarily by experts’ competence (solving or not solving clients’ problems), not morality (moral or immoral intentions). The global impressions of the experts who made a mistake and did not solve clients’ problems were negative regardless of the experts’ moral or immoral intentions. However, the competent experts were continually assessed positively regardless of their good or bad intentions. The meta-analysis showed that the effect of manipulated intention on global impression was not significant. The results pose a challenge to the idea that moral behaviors are the most relevant when making global impressions of others.

## Introduction

Imagine that your car is broken, and you need it to be fixed by tomorrow because you are planning to go on holiday. You go to your car mechanic, whose services you have used for some time, and ask for help. You know that they have reasonable prices for car repairs and put their clients’ interests before their financial benefit. Additionally, the car mechanic promises, in this case, to repair your car before other clients, without taking additional payment. However, surprisingly, he or she makes a mistake, and your car is still broken. Your holiday is ruined. What would be your global impression of the mechanic who wanted to help you but made a mistake? Would you have a positive global impression of a car mechanic who successfully repaired your car but whose main goal was not to help you but earn money by overstating the service’s price, seeing that you are in a rush? In other words, how important for you is not only the competence but also the intentions of your car mechanic?

Past findings have shown that moral behaviors and traits predominate in all stages of person perception, from the early stages of information processing to the formation of overall impressions of others (Brambilla et al., 2021; Brambilla & Leach, 2014; Ellemers, 2017; Goodwin, 2015; Goodwin et al., 2014; Landy et al., 2018). This means that when forming impressions of others, we mainly care about how moral they are, not how competent they are. It was called the primacy of morality hypothesis (Brambilla et al., 2021) or the dominance of morality hypothesis (Melnikoff & Bailey, 2018). This priority of the morality dimension in people’s evaluation of social targets is also one of the main premises of the behavior regulation model (Ellemers, 2017). Its higher social value widely explains the dominance of the morality dimension in many areas (Abele & Wojciszke, 2014).

Although some models allowed for the possibility that morality’s role may be constrained under certain conditions, they generally underestimated the possibility of a reversal. This may be because the experimental paradigms that did not take into account the real social issues and current individual goals were often used in the research (see the review: Abele & Wojciszke, 2014; Abele et al., 2021; Nicolas et al., 2021).

However, we hypothesize that there is a group of people whose specific social role makes their competence in virtually every situation predominate their morality. This group comprises those known as experts whose professional role is to use their own expertise to solve other people’s problems. Their competence is thus not (or not only) self-profitable but mainly other-profitable and, therefore, socially useful (Feltovich et al., 2006; Mieg & Evetts, 2018). The relationship with experts is one of the most fundamental types of social interactions: An asymmetrical relationship. Like parent-child, teacher-student, or supervisor-subordinate relationships, the interaction with the expert is asymmetric as the expert holds a stronger position (because of the higher knowledge in the domain) than his or her client. By testing the primacy of morality hypothesis in asymmetrical relationships, we not only test proposed models (Brambilla et al., 2021; Abele et al., 2021) but also bring those models closer to real-life interactions (Bostyn et al., 2018; Schein, 2020).

In the current research, we investigated how people make global impressions about experts based on information about their competence (solving the problem or not) and morality (moral or immoral intentions, i.e., desire to help the client vs. to make money by overstating the service’s price). We run three studies focusing on different types of experts: a financial advisor (Study 1a), a car mechanic (Study 1b), and a physician (Study 1c) to answer the question of how strongly people care about experts’ competence and morality when making global impressions about them.

## Morality and Competence – Basic Dimensions of Social Perception

Social perception research has shown that two fundamental content dimensions underlie interpersonal and group judgments (Abele & Wojciszke, 2014). Although different names are used for both dimensions, such as communion (Abele & Wojciszke, 2007), morality (Phalet & Poppe, 1997), warmth (Fiske et al., 2002), competence (Fiske et al. 2002), agency (Abele & Wojciszke, 2014), or ability (Brycz & Wojciszke, 1992), they are all described similarly. The dimension of ‘communion/morality/warmth’ signals someone’s relation to other people (related to our social and moral traits). It is also called horizontal because it indicates the willingness of targets to move closer, irrespective of hierarchy (Abele et al., 2021). The dimension of ‘competence/agency/ability’ is a signal of one’s ability to accomplish goals (it is related to skills). It is also called vertical because it indicates the hierarchical position of social targets (Abele et al., 2021). ‘morality’ is exemplified by traits such as honesty, fairness, and loyalty, whereas ‘competence’ pertains to capability, efficacy, and intelligence (Abele & Brack, 2013). It is worth noting that ‘communion’ sometimes is divided into two types: (1) the first of these (sub) dimensions defines an individual’s actions regarding the benefits or losses they can bring to others, and it is commonly named ‘morality’; (2) the second, so-called ‘sociability’, pertains to cooperation and form connections with others (Brambilla et al., 2011, Brambilla et al., 2021). In the current research, we focused on ‘morality’, not ‘sociability’, because moral attributes and behaviors are more relevant to impression formation than attributes and behaviors related to sociability (Abele & Wojciszke, 2007; Stasiuk, 2020).

## The Primacy of Morality

People usually prefer moral and competent individuals as social partners; however, there is ample evidence that morality dominates competence (and sociability) in social perception (see the reviews: Abele et al., 2021; Brambilla et al., 2021). Previous studies have shown that content related to morality is processed preferentially in earlier stages of information processing, such as recognition or categorization (Abele & Bruckmiller, 2011); attitudes towards others are based more on moral than competence traits (De Bruin & van Lange, 1999); emotional reactions to others’ morality are stronger than reactions to others’ competence (Wojciszke & Szymkow, 2003); and, according to the primacy of morality hypothesis, moral traits are the most substantial contributor to – and when positive, always contribute positively to – global impressions of others (Landy et al., 2018, Brambilla et al., 2021).

The explanations of this widely observed ‘power of morality’ are usually grounded in a functional approach to person perception, according to which ‘perceiving is for doing,’ and the primary purpose of social perception is to guide people in their actions (Fiske et al., 2007). Individuals with positively assessed morality (including moral intentions) can be safely approached, while those with negatively assessed morality should be avoided. According to this explanation, the individual’s competence is of only minor importance to others’ perceptions (Wojciszke, Bazinska, Jaworski, 1998). It was assumed that information about others’ morality determines their global evaluation’s valence, while information about others’ competence can only change the intensity of this evaluation within the valence. A person behaving immorally was assessed negatively even if they were highly competent, while a person behaving morally was assessed positively even if they lacked competence (Wojciszke, Bazinska, Jaworski, 1998).

Despite the overwhelming number of results proving the dominance of morality in social perception, several researchers point out that in some situations, the role of morality has decreased. In several experiments, Melnikoff and Bailey (2018) questioned the assumption that moral traits always contribute positively to one’s global impressions of others. They showed that the liking of people who possess moral and immoral traits depends on one’s current goals, and sometimes, people prefer immoral others over moral others. More specifically, they found across four studies (using both explicit and implicit measures) that when morality was goal-conducive, moral traits increased liking, but when immorality was goal-conducive, the preference for moral traits was eliminated or reversed. For example, not all participants liked the honest (moral) spy more than the dishonest (immoral) spy. The liking of the spy was conditional, and it depended on whether participants had benefited from the spy’s honesty. However, as correctly pointed out by Landy and colleagues (2018), Melnikoff and Bailey (2018) focused on liking (i.e., warm feelings), not global impressions, so that is why they still concluded that morality traits always dominate in forming impressions of others.

The role of an individual’s goals in making impressions on others was also highlighted by Abele and Wojciszke (2014) in their dual perspective model. They pointed out that the kind of relationship may be a moderating factor for the interest in another’s agency and morality (Abele & Wojciszke, 2014). Although social judgments are typically dominated by communal content, in exchange relationships, an actor’s competence receives increased weight from the observer’s perspective, as it becomes crucial for the observer’s goals. In our studies, we sought to test further the assumption that the morality dimension is not always the ‘strongest contribution to global impressions of others’ (Landy et al., 2018). We planned to do so by showing that there are important cases when morality loses its absolute power to competence. We expected that these specific circumstances would be associated with the perception of experts.

## Hypotheses

Experts are distinguished from others by specific knowledge or skills in a particular field (Feltovich et al., 2006). Experts are those who not only have more knowledge and skills than laypeople but also (or above all) have a unique role in society. This role is to create specialized knowledge and share it with people who need help solving certain problems in a specific field (Mieg & Evetts, 2018). Because an expert’s role is to solve the problems of clients who do not have enough knowledge or skills to do it alone, the characteristics and behaviors that are most desirable for experts should be those related to competence, as they allow experts to achieve the target’s goals. This assumption can be further justified by the abovementioned role that experts play in the social structure. The criteria for considering someone as an expert are knowledge or skills (confirmed formally or assigned by others), so the competence dimension is the basis for an expert’s definition and should be more critical than the expert’s morality. It means that in the case of experts, the pattern of results obtained in the research mentioned above on social perception should be reversed.

We hypothesized that the expert’s competence, not moral intentions, is the most substantial contributor to their global impression and determines its valence. Experts who do not solve clients’ problems (low competence) would be therefore evaluated negatively, even if their intention was moral (e.g., because they had good intentions and wanted to help the client). Experts who solve clients’ problems (high competence) would be evaluated positively even if their intention was immoral (e.g., because their intention was not to help the client but to make money by overstating the service’s price). We focused on experts’ moral and immoral intentions because these are commonly used in research as a signal of someone’s morality (e.g., Cushman, 2008). People’s morality informs us about their likely intentions, and higher morality evaluations (or moral character evaluations) are related to good intentions and *vice versa* (Landy et al., 2015).

## Overview of the Studies

We ran three experiments with *N* = 413 participants where we used similar procedures, asking the participants to read fictional scenarios about experts. We manipulated information about experts’ intentions (moral/good or immoral/bad) and effectiveness (solved clients’ problems or not). To test our hypothesis, we studied the perception of three types of experts: a financial advisor (Study 1a), a car mechanic (Study 1b), and a physician (Study 1c).

The decision to use the abovementioned types of experts was made based on a pilot study. First, we presented the definition of an expert to *N* = 30 participants, and then we asked participants to list five categories of experts that came immediately to their mind and whose services they used most often. The most common types of experts were physicians, lawyers, financial advisors, car mechanics, plumbers, and architects. Next, we conducted a survey asking *N* = 300 participants about six experts. The questions concerned whether participants often take advice from these experts. The results helped us choose the three most popular experts: physicians, financial advisors, and car mechanics, which were used in a randomized order in the current research. The data and study materials (the used scenarios) are available at https://osf.io/r4pbv/?view_only=None.

Since we used analogous procedures and measurements (only the content of the scenario changed according to the expert’s profession), we present below a joint description of the method and results for the three experiments.

## Method

### Participants

Power analyses were performed with G*Power 3.1.9.6 (Faul et al., 2009) to determine the sample size. Sample sizes were calculated under the following assumptions: alpha = .05; power = 80%; and small, medium, and large effect sizes: Cohen f = .10, .25, and .40. The required sample sizes for the main effects and interactions were 787, 128, and 52. Given the constraints, sample sizes of approximately 130–140 participants were planned, allowing for reasonable power to detect medium and large effect sizes.

In Study 1a, we recruited *N* = 134 Polish participants (*M*_age_ = 31.5, *SD* = 10.2, range: 19–57; *n* = 79 females) through social media announcements and university websites for no monetary compensation. No gender differences were found concerning age or experimental conditions.

In Study 1b, we recruited 141 Polish participants (*M*_age_ = 31.5, *SD* = 11.3, range: 19–67; *n* = 82 females) with the same procedure used in Study 1. No gender differences were found concerning age or experimental conditions.

In Study 1c, we recruited 138 Polish participants (*M*_age_ = 42.7, *SD* = 11.6, range: 22–68; *n* = 82 females) using the same procedure as in the previous studies. Again, no gender differences were found concerning age or experimental conditions.

### Procedure and Measures

The studies were run online via the Google Forms platform. The participants in each study were randomly assigned to one of four conditions in a between-group design. Participants were asked to read a scenario of a client’s visit to a financial advisor (Study 1a), car mechanic (Study 1b), and physician (Study 1c).

In Study 1a, the client wanted to buy an apartment and was determined to get a mortgage as soon as possible. In Study 1b, the respondents read a scenario where a client whose car had broken down before going on vacation arrived at the mechanic’s shop and asked the mechanic to repair the car quickly. In Study 1c, the participants were presented with a scenario where a patient visited a physician complaining about severe leg pain caused by varicose veins and asked him to perform the procedure quickly.

The independent variables in each study were information about the effectiveness of the action taken by the expert to solve the client’s problem and information about the expert’s intention in taking this action.

In Study 1a, participants were told that the advisor made a mistake in filling in the forms, that the client did not get a mortgage (low competence), or that the expert arranged the mortgage efficiently and quickly (high competence). They were also told that the advisor was guided mainly by his profit (immoral intention) or willingness to help the client (moral intention).

In Study 1b, participants were presented with information that the expert made a mistake, and the car was still broken vs. the car was successfully repaired (high vs. low competence) and with information about the expert’s intention (desire to help the client vs. to make money by overstating the service’s price).

In the Study 1c, we again manipulated the effectiveness of the actions taken by the expert (they operated on the patient, and the patient already feels better vs. they made a mistake, and the patient will have to undergo the procedure again) and the intentions of the expert (they decided to perform the procedure quickly because they wanted to help the patient vs. primarily because they wanted to earn money).

After reading the scenario, the respondents were asked how moral and competent the expert is and a general evaluation of him/her. Three questions assessed the expert’s competence (‘How do you assess the expert’s skills?’, ‘How do you assess the expert’s knowledge?’, and ‘How do you assess the expert’s effectiveness?’) rated on a scale from –5 to 5, where –5 means *definitely bad* and 5 means *definitely good*. The answers to these questions were averaged to form one factor, ‘competence’. Three other similarly constructed questions referred to the dimension of morality (‘How do you assess the expert’s truthfulness?’, ‘How do you assess the expert’s honesty?’, and ‘How do you assess the expert’s morality?’). These questions were averaged to form a ‘morality’ factor’. The last three questions concerned the general evaluation of the expert (‘How do you generally evaluate this expert?’, ‘Would you go to this financial advisor/car mechanic/physician if you have financial problems/problems with car/problems with health?’, ‘Would you recommend this expert to others?’). The respondents answered on the same scale as the previous questions. The answers were averaged to form a ‘Global Impression’ factor.

Cronbach’s alphas for the three factors mentioned above, competence, morality, and global impression, were calculated separately for three experiments. For financial advisor (Study 1a), Cronbach’s alphas were: 885 for competence, 912 for morality, and 932 for global impression. For car mechanic (Study 1b): 923 for competence, 924 for morality, and 928 for global impression. For physician (Study 1c): 966 for competence, 948 for morality, and 953 for global impression. Next, gender differences in the dependent variables were tested, with non-significant results.

## Results and Discussion

The primary analyses were performed with PS IMAGO 25 software. As we mentioned in the hypotheses section, we wanted to investigate how the expert’s effectiveness and intentions affect their global impressions. Initially, we treated the evaluation of morality (Morality factor) and competence (Competence factor) only as a manipulation check. However, after analyzing the results, we found that information about experts’ mistakes or effectiveness affected the evaluation of their competence (which we assumed) and changed the evaluation of their morality (Morality factor). Conversely, information about the expert’s intentions influenced the evaluation of their morality (Morality factor) and their competence (Competence factor). We found these results to be important not only from the theoretical but also from the practical point of view. Therefore, we decided not only to provide information about the effectiveness of the manipulation but also to describe all the results related to the evaluation of both dimensions. In the **Results** section, we first present the results of the manipulation check, then we provide information about experts’ global impressions (as this was the main objective of the study), and we then describe the analyses in which manipulated effectiveness and intention (and their interaction) were predictors, the assessed competence and morality of the expert were mediators, and global impression was the outcome. Lastly, we present a meta-analysis.

### Manipulation Check for Studies 1a (Financial Advisor), 1b (Car Mechanic), and 1c (Physician)

#### Financial Advisor

The main effect of information about the financial advisor’s effectiveness in arranging the mortgage on his/her perceived competence was significant: the competence of financial advisors who made a mistake was rated negatively (*M* = –0.36), while advisors who did not make a mistake received positive ratings of competence (*M* = 3.69; *F*(1, 130) = 124.49, *p* < .001, *η^2^* = .49). The effect size was relatively large. The main effect of information about the expert’s moral/good or immoral/bad Intention on his/her perceived morality was also significant: the financial advisors who acted for profit were rated lower on morality (although still positively) than those acting to help their clients (*Ms* = 0.12 *vs*. 2.85, *F*(1, 130) = 59.13, *p* < .001, *η^2^* = .31).

#### Car Mechanic

The main effect of the car mechanic’s effectiveness on repairing the client’s car was significant and strong: car mechanics who made a mistake were rated lower (and negatively) in terms of competence than those who repaired the car (*Ms* = –0.72 *vs*. 3.53; *F*(1, 137) = 136.15, *p* < .001, *η^2^* = .50). The effect of intention was also significant: car mechanics who acted for money were rated lower on morality (and negatively) than those acting to help their clients (*Ms* = –0.16 *vs*. 2.30, *F*(1, 137) = 30.80, *p* < .001, *η^2^* = .18).

#### Physician

The main effect of information about physician’s effectiveness was significant and very strong: the competence of physicians who made a mistake was rated lower (and negatively) than that of those who did not (*Ms* = –1.57 *vs*. 3.53; *F*(1, 134) = 150.85, *p* < .001, *η^2^* = .53). The effect of information about physician’s intention on the evaluation of morality was also significant and very strong: physicians who acted for profit were rated negatively and much lower on morality than those acting to help their patients (*Ms* = –2.39 *vs*. 2.32, *F*(1, 134) = 126.88, *p* < .001, *η^2^* = .49).

### The Global Impression of Financial Advisor (Study 1a), Car Mechanic (Study 1b), and Physician (Study 1c)

#### Financial Advisor

The main effect of effectiveness was significant and robust: the global impression of financial advisors who made a mistake was lower (and negative) than that of those who did not make one (*Ms* = –1.61 *vs*. 2.59; *F*(1, 130) = 106.10, *p* < .001, *η^2^* = .49). The effect of intention was also significant, although less sizeable; the global impression of financial advisors who acted for profit was lower (but not significantly different from 0) than that of those who wanted to help their clients (and who were evaluated positively, *Ms* = –0.17 *vs*. 1.54, *F*(1, 130) = 21.01, *p* < .001, *η^2^* = .14).

To analyze the difference in the effect sizes between the two main factors (Effectiveness and Intention), they were expressed as beta coefficients (with the interaction term included in the multiple regression), and the standard formula for determining the significance of the differences was applied, that is, the difference in estimates was divided by the standard error of the difference. The specific formula was used: Standardized difference = (Beta1 – Beta2) / sqrt (Var (Beta1) + Var (Beta2) – 2* Cov (Beta1, Beta2). The difference between effects was considered significant when the standardized difference was greater than 2.00.

**Figure 1:**
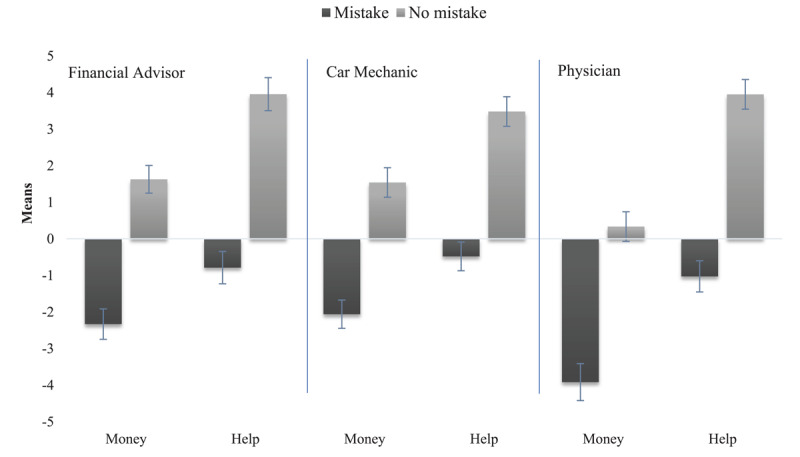
Means and Standard Deviations Across Experimental Conditions in Studies 1a, 1b, and 1c (Dependent Variable: Global Impression). Path analyses for Financial Advisor (Study 1a), Car Mechanic (Study 1b), and Physician (Study 1c).

The difference in effect sizes between the factors effectiveness and intention proved statistically significant, as their standardized differences exceeded 2.0 (it was 4.43). It must be noted that these results support our prediction. The global impression of financial advisors who made a mistake was negative, regardless of the intention, and the global impression of financial advisors who did not make a mistake was positive, even when they acted for their benefit. Finally, the interaction of effectiveness with intention was not significant (*F*(1, 130) = 0.86, *p* = .355, *η^2^* = .01). Means and standard deviations across experimental conditions in Study 1a for the dependent variable Global Impression of the financial advisor are in Figure 1.

#### Car Mechanic

The main effect of effectiveness was significant and strong: the global impression of car mechanics who made a mistake was lower than the overall impression of those who did not. The effect size was large (*Ms* = –1.28 *vs*. 2.51; *F*(1, 137) = 90.76, *p* < .001, *η^2^* = .40). The effect of intention was also significant, although less sizeable; the global impression of car mechanics acting for profit was lower (but not significantly negative) than that of mechanics acting to help their clients (*Ms* = –0.34 *vs*. 1.44, *F*(1, 137) = 19.68, *p* < .001, *η*^2^ = .13). The difference between the effect sizes for effectiveness and intention was significant (standardized difference = 4.04). Again, these results support our prediction. The global impression of car mechanics who made a mistake was negative, regardless of the intention, and the global impression of car mechanics who did not make a mistake was positive, even when they acted for their benefit. The effect of effectiveness on the global impression was not significantly moderated by intention (*F*(1, 137) = 0.21, *p* = .651, *η*^2^ < .01). Means and standard errors across experimental conditions in Study 1b for the dependent variable global impression of the car mechanic are in Figure 1.

#### Physician

The global impression of the physician who made a mistake was negative and lower than the global impression of those who did not make a mistake. The effect size was large (*Ms* = –2.23 *vs*. 2.14, *F*(1, 134) = 111.34, *p* < .001, *η^2^* = .45). The effect of intention was also significant: the global impression of physicians acting for profit was also negative and lower than the global impression of those actions with the Intention to help their patients. The effect size was also large but smaller than the effect size for effectiveness (*Ms* = –1.33 *vs*. 1.59; *F*(1, 134) =55.32, *p* < .001, *η^2^* = .29). The effectiveness effect size was significantly larger than that for Intention (standardized difference = 2.49). As in Studies 1a and 1b, these results support our prediction. The global impression of physicians who made a mistake was negative, even when they intended to help the patient, and the global impression of physicians who successfully performed the procedure was positive, even when they acted for their benefit.

The effect of competence on global impression was not significantly moderated by morality (*F*(1, 134) = 0.68, *p* = .409, *η^2^* = .01). Means and standard errors across experimental conditions in Study 1c for the dependent variable global impression of the physician are presented in Figure 1.

**Figure 2:**
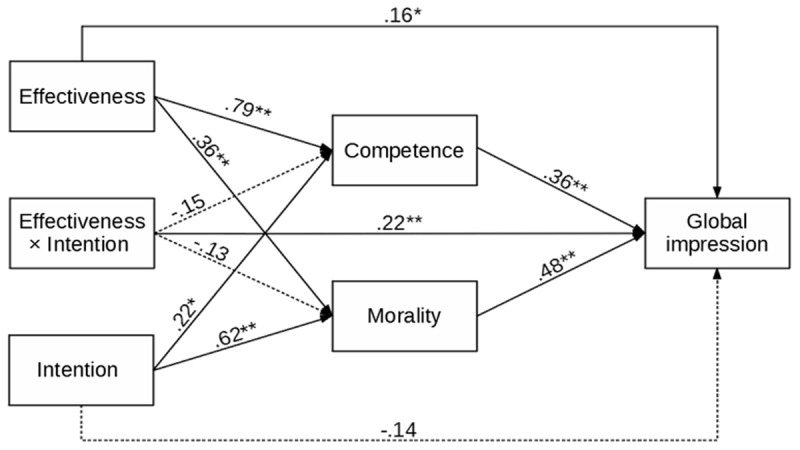
Study 1a – Results of the Path Analysis for Global Impression of Financial Advisor. *Note*: ** *p* < .001, * *p* < .05.

**Figure 3:**
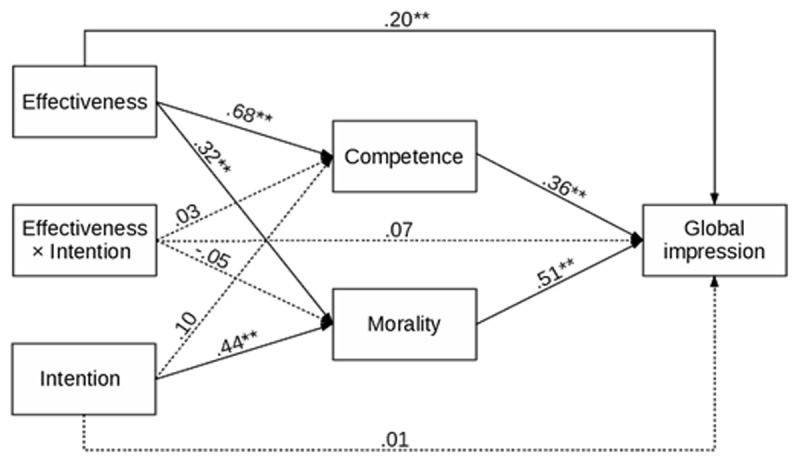
Study 1b – Results of the Path Analysis for Global Impression of Car Mechanic. *Note*: ** *p* < .001, * *p* < .05.

**Figure 4:**
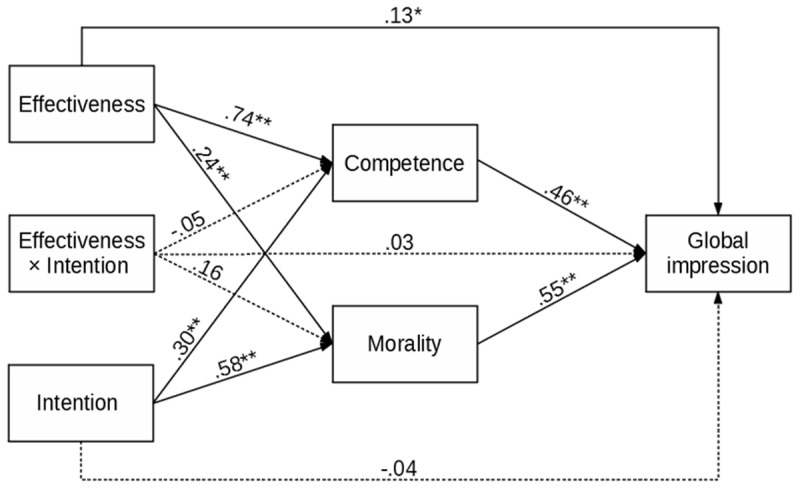
Study 1c – Results of the Path Analysis for Global Impression of Physician. *Note*: ** *p* < .001, * *p* < .05.

To further strengthen our point, we ran separate path analyses for Studies 1a, 1b, and 1c. In each analysis, the manipulated effectiveness and intention (and their interaction) were predictors, the assessed competence and morality of the expert were mediators, and global impression was the outcome. Such a model makes it possible to test the impact of measured competence and morality on global impression, which is at the core of our hypothesis. Indirect effects of manipulated effectiveness and intention and their interaction on global impression were also assessed. The results are presented in Figures 2, 3, 4, and Tables 1, and 2.

**Table 1:** Studies 1a,1b,1c – Indirect Effects in the Path Analyses for Global Impression of Experts.


PREDICTOR	MEDIATOR	FINANCIAL ADVISOR				CAR MECHANIC				PHYSICIAN			
		
		*BETA*	*SE*	*Z*	*P*	*BETA*	*SE*	*Z*	*P*	*BETA*	*SE*	*Z*	*P*

Effectiveness	Perceived competence	.28	0.37	4.88	<.001	.25	0.30	4.86	<.001	.34	0.38	6.34	<.001
	
Effectiveness × Intention		–.06	0.29	–1.47	.142	.01	0.25	.32	.749	–.02	0.37	–.46	.645
	
Intention		.08	0.22	2.27	.023	.04	0.18	1.15	.251	.14	0.30	3.25	.001

Effectiveness	Perceived morality	.18	0.31	3.59	<.001	.16	0.32	2.97	.003	.13	0.34	2.72	.006
	
Effectiveness × Intention		–.06	0.43	–1.12	.264	–.03	0.44	–.43	.668	.09	0.45	1.52	.128
	
Intention		.30	0.38	5.06	<.001	.22	0.33	4.03	<.001	.32	0.39	5.63	<.001


*Note*: *Beta*: standardized path coefficients; *SE*: standard errors; *Z: Z* statistics; *p: p* values for the significance of the paths.

To get a synthesis of the results of all three experiments, a set of meta-analyses was performed. Each effect represents a respective ‘path’ from the model, calculated jointly for all three studies. As suggested by Peterson and Brown (2005), Beta coefficients were entered as correlations. As the effects might differ across studies, random effects were computed with 95% confidence intervals. Software Comprehensive Meta-Analysis 2 was used (Borenstein et al., 2006). The results are presented in Table 2.

**Table 2:** Meta-analysis Across Three Experiments (Financial Advisor, Car Mechanic, Physician).


PREDICTOR	DEPENDENT	*EFFECT*	*LLCI 95%*	*ULCI 95%*

Effectiveness	Competence	.77	.73	.81

	Morality	.35	.26	.43

Intention	Competence	.25	.14	.36

	Morality	.59	.48	.68

Effectiveness	Global Impression	.21	.12	.30
	
Intention		–.04	–.15	.07

Competence	Global Impression	.44	.35	.51
	
Morality		.55	.48	.62

Effectiveness × Intention	Competence	–.04	–.17	.09

	Morality	.10	–.06	.24

	Global Impression	.15	.04	.26


*Note*: LLCI, UCLI: 95% confidence intervals.

Study 1a showed that financial advisors who acted ineffectively (made a mistake and did not solve the client’s problem) were rated negatively, while financial advisors who acted effectively (solved the client’s problem) were rated positively. The information about their intentions (moral or immoral) only changed this positive or negative evaluation’s intensity. The results of the path analysis confirm and extend the results of the analysis of variance. The impact of the effectiveness manipulation was significant and positive not only for assessed competence but also for morality. The impact of manipulated intention was also significant and positive for assessed competence and morality. The direct effects of manipulated competence on global Impression were significant, but the effect of manipulated intention was not. The impact of competence and morality on global impression was significant. Importantly, the indirect effects of effectiveness and intention on global impression were significant. Finally, the only significant effect of the interaction of effectiveness and intention was its impact on global impression.

The pattern of the results obtained in Study 1b was the same as those obtained in Study 1a. Car mechanics who made a mistake and did not solve the client’s problem were evaluated negatively, while car mechanics who were able to solve the client’s problem were evaluated positively. The information about their intentions only changed the intensity of positive or negative evaluations. The results of path analysis were similar to Study 1a. The impact of effectiveness was positive and significant for both competence and morality, as well as global impression. The effect of intention was significant for morality but not for competence (nor global impression). The effects of competence and morality on global impression were positive and significant. The interaction between effectiveness and intention was not significant for any of the variables. The indirect effects of effectiveness on global impression were significant. The indirect effect of intention via morality (not competence) on global impression was significant. Finally, the mediation for the interaction of effectiveness and intention was not significant.

In Study 1c, we replicated the findings from Study 1a and Study 1b, showing that making a mistake contributed to the experts’ overall impressions (here, physicians). Information about the physician’s intention changed only the overall impression intensity and not its valence (determined by effectiveness). The results of path analysis were similar to previous studies. The impact of effectiveness was positive and significant for competence, morality, and global impression. The effect of intention was also significant for morality and competence but not for global impression. The effects of competence and morality on global impression were positive and significant. The interaction between effectiveness and intention was not significant for any of the variables. Importantly, the indirect effects of effectiveness and intention on global impression were significant. Finally, the mediation for the interaction of effectiveness and intention was not significant.

The meta-analysis showed that the manipulation with effectiveness was significant and positive for assessed competence and morality. The impact of manipulated intention was also significant and positive for both assessed competence and morality. The direct effects of manipulated competence on global impression were significant. Most importantly, the effect of manipulated intention on global impression was not significant. The effects of assessed effectiveness and were both positive and significant. As for the interaction between effectiveness and intention, it was only significant in the case of global impression.

## General Discussion

Evaluation of other people is a part of our everyday life. Several psychological models try to explain how it happens and when we see others in a more positive or more negative light (see the reviews: Abele et al., 2021; Brambilla et al., 2021) and how this evaluation depends on our current goals (Nicolas et al., 2021). Our research was based on the dual perspective model (Abele & Wojciszke, 2007) and the moral primacy model (Brambilla et al., 2021) and tested the primacy of morality hypothesis. The hypothesis has two critical assumptions: that moral intentions, behaviors, or traits are the most substantial contributors to others’ global impressions and that moral intentions, behaviors, and always attributes positively contribute to these global impressions (Brambilla et al., 2021; Landy et al., 2015; Landy et al., 2018). Although this hypothesis has strong supporting evidence, we found an area where it can be partially challenged: business and services, namely, the relationship between clients and experts.

We claim that the hypothesis may be partially challenged because our research confirms only its second assertion. As in previous research, information about the experts’ positive intentions in our three experiments contributed positively to the global impressions, whereas information about their unfair intentions contributed negatively. However, none of our three studies confirmed the first assumption that moral/good vs. immoral/bad intentions directly determine the general evaluation. When participants were asked to form a global evaluative impression of experts based on information about their effectiveness and intentions, they were guided primarily by the first of these dimensions. The global impression of the experts who made a mistake was always negative; the respondents would not use their services and would not recommend them to others. In contrast, the experts who solved the client’s problem without complications were continually assessed positively, and the respondents declared a willingness to use their services and were inclined to recommend them to others.

The information about the experts’ intentions (willingness to help the client vs. focusing on financial benefit and overstating the service’s price), which referred to the moral dimension, also significantly influenced participants’ overall impressions of them. However, morality information changed only the intensity of this impression, not its valence. Therefore, the overall impressions of the experts who made a mistake were generally negative but less negative for those who wanted to help the client than those who acted for financial benefit. The overall impression of experts who effectively solved the client’s problem was positive, but it was less favorable for those who acted in self-interest. We replicated our findings with different experts, showing that the specific expert area is not highly relevant to impression formation. We also found no gender differences, consistent with previous findings that moral information determined global impressions more strongly than warmth for both men and women (Goodwin et al., 2014). It is worth highlighting that we did not prove that morality is irrelevant to experts’ perceptions. It is just not the main component of their global impressions.

Although not fully supported by the previous studies, our results can also be explained by referring to perception’s functionality. According to the functionalist standpoint, when we evaluate others, this evaluation is determined by their behavior and characteristics that are beneficial to us (Fiske et al., 2007; Abele & Wojciszke, 2014). We usually benefit more from others’ morality than from competence, and the immorality of others harms us more than their incompetence. However, this is not the case with experts. First and foremost, we turn to experts when solving our problem requires knowledge or skills, not to have ourselves. Therefore, we benefit more from their ability to solve the problem in the given domain (relevant to our problem) than from their moral/good intentions and behaviors, and their mistakes can usually do us more harm than their immoral/bad actions. This explanation is also consistent with the finding that the current individual’s goals determine which dimension (i.e., competence, morality, or sociability) is the most important in the social perception process (see: Abele & Wojciszke, 2014; Nicolas et al., 2021).

In practice, our results do not mean, of course, that an expert who is able to solve the client’s problem effectively can afford to express immoral intentions without negative consequences (e.g., legal or social), mainly when operating in a market where competition is high. When presented with the choice between a physician who can quickly make the correct diagnosis and prescribe the proper treatment but whose intention is only financial profit and a physician who is equally competent but considered to have moral intentions, most patients will probably choose the latter.

The analysis of the results also revealed the influence of information about the experts’ mistakes vs. effectiveness in evaluating their morality and the influence of information about the experts’ intentions to evaluate their competence. The morality of experts who made a mistake was evaluated significantly lower than that of experts who did not make a mistake. It could be assumed that the expert’s mistake was due to neglect of the client’s problem and the insufficient investment of effort or attention to solve it. However, in the presented texts, in the case of each of the experts (even those whose intention was to make money), there was clear information that before taking action, they precisely assessed the situation of the client/patient (the physician thoroughly examined the patient, the mechanic thoroughly checked the car, and the advisor thoroughly familiarized him/herself with the financial situation of the client). Moreover, the evaluation of the experts’ competence appeared to be influenced by information about their morality. Their knowledge, skills, and effectiveness were rated lower when they were solving the client’s problem for their profit than when they were doing it primarily to help him or her.

It is possible that this influence of information in one dimension (e.g., competence) on the evaluation of characteristics in another dimension (e.g., morality) is a consequence of the halo effect (Fisicaro & Lance, 1990). The halo effect is the spread of evaluations of a person’s perceived trait to other traits. If we consider someone friendly based on their behavior, it will result in positive evaluations of their morality or intelligence, even if there is no clear indication that this is justified. The halo effect has long been known in psychology and is explained by different models (Fisicaro & Lance, 1990; Gräf & Unkelbach, 2016). The classic global impression model assumes that general target impressions influence attribute ratings; the common variance source creates correlations among attributes. This is referred to as the indirect halo effect. The salient dimension model assumes that information about salient features directly influences other attribute ratings, which is described as the direct halo effect. As the dimensions of competence and morality are theoretically orthogonal, our results can probably be explained by an indirect halo effect, which should be apparent across dimensions, rather than a direct halo effect (which should occur within dimensions – from competency/morality behaviors and traits to another competency/morality behaviors and traits). Based on the information provided, our participants formulated an overall assessment that the expert is a good (or bad) physician, mechanic, or financial advisor, which, in turn, influenced the assessment of her/his competence and morality. Since the information about the expert’s mistake or effectiveness is more critical for the overall assessment, the impact of this information on the evaluation of morality was more substantial than the impact of information about intention on the evaluation of competence.

Our result is in line with the previous studies showing a positive relationship between two dimensions when the single social object was judged, whereas the compensation effect appeared when a comparison was made between two social objects (Judd et al., 2005). The positive relationship between competence and morality can also be explained by referring to the cooperative nature of the relationship between the client and the expert (as they work together to solve the client’s problem). As the studies of Carrier, Dompnier, and Yzerbyt (2019) showed, when the perceiver (here: client) had to collaborate with a target (here: expert), the more the target came across as competent, the more the perceiver attributed warmth to the target. The opposite pattern was found in the competition domain.

Another explanation of our results can be found in work ethic, which is a system of values or beliefs related to work itself and strongly impacts workers’ behaviors (Grabowski et al., 2019). Works ethics consists of the centrality of work (i.e., treating work as a central part of life), self-reliance (i.e., striving for independence in one’s daily work), hard work (i.e., belief in the virtues of hard work), leisure (i.e., disapproval of leisure activities), morality (i.e., believing in justice and that people should be fair when making their moral decisions), delay of gratification (i.e., orientation toward the future, the postponement of rewards), and unwillingness to waste time (i.e., beliefs reflecting active and productive use of time) (Miller et al., 2002). It can be assumed that the distinction between competence and morality (Abele & Wojciszke, 2007) in the professional domain is somewhat reductive, given that expert competence can be considered a form of morality. Indeed, the concept of work ethic can lead to a perception of a person who competently performs his or her job as moral. Conversely, serving the client’s interest and taking care of the client’s needs can be seen as an aspect of competence.

The influence of information in one dimension (e.g., about effectiveness) on the evaluation of characteristics in another dimension (e.g., morality), as well as the concept of work ethic and the interpenetration of the dimensions of competence and morality in the case of experts, may explain the results that an experts’ perceived morality influenced overall impression similarly to their perceived competence.

Although our findings provide compelling evidence about the limitations of the primacy of morality hypothesis, it seems appropriate to acknowledge limitations. First, we used the information about the expert’s intentions to evaluate morality. We focused on experts’ moral or immoral intentions because these are commonly used in research to signal someone’s morality (e.g., Cushman, 2008). People’s morality informs us about their likely intentions, and higher morality evaluations (or moral character evaluations) are related to moral intentions and *vice versa* (Landy et al., 2015). However, future research could focus on the harm the expert has done to the client intentionally, following the Knobe effect (Knobe, 2003) or the findings showing that directly harming someone is perceived as morally worse than failing to help them (Baron & Ritov, 1994). Second, we focused only on three types of experts. Future studies could generally add other types of experts or other social groups, looking for other limitations of the primacy of morality hypothesis.

The results of the present studies appear to have important theoretical and practical implications. First, we showed that the primacy of morality hypothesis has boundaries. Besides, our results contribute to understanding what laypeople expect from experts, which is particularly important given the current palpable mistrust of experts and a tendency to dismiss their advice (Nichols, 2017; Stasiuk et al., 2016). Looking for our results’ practical implications, we recommend focusing on experts’ competencies as the competencies are substantial in experts’ global impressions.
